# Awareness regarding risk factors of colon cancer and practices regarding screening for CRC among population of Aljouf region 2022, Kingdom of Saudi Arabia

**DOI:** 10.12669/pjms.41.4.11192

**Published:** 2025-04

**Authors:** Hafsa Raheel, Faisal Alazmi, Mohammed S H Aloufi, Hamad Aljubayr, Mohammed Alhaqbani, Mohammed S Alawi

**Affiliations:** 1 Hafsa Raheel Department of Family Medicine, King Saud University, Riyadh, Saudi Arabia; 2 Faisal Alazmi Department of Family Medicine, King Saud University, Riyadh, Saudi Arabia; 3 Mohammed S H Aloufi Department of Family Medicine, King Saud University, Riyadh, Saudi Arabia; 4 Hamad Aljubayr Department of Family Medicine, King Saud University, Riyadh, Saudi Arabia; 5 Mohammed Alhaqbani Department of Family Medicine, King Saud University, Riyadh, Saudi Arabia; 6 Mohammed S Alawi Department of Family Medicine, King Saud University, Riyadh, Saudi Arabia

**Keywords:** Awareness, Colorectal cancer, Colon cancer, Knowledge, Risk factors

## Abstract

**Background & Objective:**

According to the Saudi Cancer Incidence Report 2020, colon cancer ranks number one in terms of incidence in the country. To assess awareness about its prevention, we aimed to assess the level of awareness and screening practices.

**Method:**

A cross-sectional study was conducted in the three governorates of Aljouf region, Sakaka, Tabarjal, and Qurayyat, one mall and one souq (bazaar) were randomly selected in each of these to cover all different social groups. We surveyed 422 participants using a structured questionnaire to gather information regarding socio-demographic characteristics, general knowledge about colon cancer and its signs and symptoms, and risk factors of colon cancer.

**Results:**

While 71% of participants believed that early screening lowers the risk of colon cancer, more than half of respondents (52.8%) demonstrated inadequate knowledge about the signs and symptoms of colorectal cancer.

**Conclusion:**

The knowledge regarding colorectal cancer needs to be raised in the general population.

## INTRODUCTION

The age standardized incidence for cancers in Kingdom of Saudi Arabia according to data from the Saudi Cancer Incidence Report 2020 was 74.7/100,000 for males and 92.1/100,000 for females. Colorectal cancer ranked number one in males and third in the female adult population in the Kingdom.^1^ A royal decree has classified cancer as an obligatory modifiable disease due to the high occurrence of the disease in the nation. This supports precise data gathering and reporting to develop and put into action suitable preventive measures. Studies conducted in the Kingdom have reported various rates of knowledge regarding risk factor awareness. Alarfaj, 2016 reported 21% of his respondents in AlHassa region knowing that men are not more likely to get CRC than women, and 19% stated positive family history as a risk factor.

In addition, only 12% of participants correctly identified polyps as a risk factor for CRC, while 14% identified type two diabetes as a risk factor. However, a significant percentage knew that CRC occurs mainly after the age of 50. Two out of three respondents (61.5% and 34.6%, respectively) correctly identified smoking and obesity as risk factors. Over a quarter of respondents knew that physical inactivity (27.6%), excessive intake of red meat (26.2%), and fewer intakes of fruits and vegetables (27.3%) contributed to colon cancer risk.^2^

Another study conducted in Saudi Arabia, reports a general lack of knowledge about CRC risk factors and screening tools. In that study, only one-third of respondents were able to correctly define CRC, and 18% correctly identified colorectal polyps and red meat as risk factors).^3^ A study conducted in the capital city of Saudi Arabia showed marked deficiency of correct knowledge regarding risk factors for Colon cancer and screening tools.^4^ Colon cancer being a preventable non communicable disease, warrants for knowledge promotion regarding its prevention with smoking avoidance, fiber rich diets, and exercise. Increased awareness brought people to adopt early screening practices as observed in the literature.^5^ As there is paucity of studies in the literature, identifying the gap we aimed at assessing the knowledge towards CRC and their practice about CRC screening among adult population settled in Aljouf region.

## METHODS

A cross-sectional study was carried out in Aljouf region, Saudi Arabia. Aljouf region is one of the 13 regions in the Kingdom of Saudi Arabia located in north-central Saudi Arabia on the border with the Hashemite Kingdom of Jordan. With a population of more than half a million people, and three governorates covering the entire region.^6^ We conducted this study in three governorates of Aljouf region, Sakaka, Tabarjal, and Qurayyat governorate by selecting one mall and one souq (bazaar) in each of these areas, to cover all different social groups. The data was collected during October - December 2022. Data was obtained by self-administered questionnaire, that was answered by visitors of Aljouf Mall and Souq Aljouf Alshaabe regarding Sakaka district, visitors of Pyramid mall and souq Alfaris regarding Tabarjal governorate, and visitors of Qurayat mall and Souq Sakhar regarding Qurayyat governorate. Considering the fact that data from Aljouf region specially, regarding colon cancer is not readily available, we decided to conduct this research in this region.

### Ethical statement:

This study was approved by the “Institutional Review Board, College of Medicine, King Saud University, Riyadh, Saudi Arabia” (No. E-22-7204; dated October 13, 2022).

### Study population:

Adult males and females visiting the malls and souqs, greater than 30 years, living in Aljouf region for at least 10 years, were given the questionnaire. The required sample was calculated using a single proportion formula, A p-value of <0.05 and 95% Confidence. The estimated sample was 384, we added 10% to compensate for the non-response, hence a total of 422 were surveyed. This is a self-funded study, a paper with a barcode image was placed on all mentioned markets in the three governorates of Aljouf region. We asked market visitors to participate in our research. To ensure that participants were selected based in our inclusion criteria, the questionnaire was automatically modified to not completing the questions if the participant did not comply with our inclusion criteria. After they filled the survey, we provided them with a link about colon cancer in order to raise awareness**.** A pilot study was carried out on 10% of the total sample size.

### Eligibility criteria:

Male and female residents of Aljouf region since more than 10 years, over the age of 30, were enrolled. Those working in the medical field, and diagnosed with any type of cancer were excluded.

### Questionnaire:

The questionnaire consisted of demographic information, risk factors, signs and symptoms and practices regarding colorectal cancer screening. The questionnaire was constructed from literature review, and other literature on the subject.^7^-^10^

### Statistical analysis:

Statistical Package for the Social Sciences (SPSS) version 24.0 was used to conduct the statistical analysis (SPSS Inc., Chicago, IL, USA). Descriptive tables were prepared. We used Chi-square test (χ2) to compare between each of knowledge of symptoms and signs of disease group and awareness of colon cancer with respect to all demographic characteristics’ variables.

## RESULTS

The demographic characteristics of the participants are shown in Table I. Of the 422 participants, 59.7% were male, 63.7% were aged between 30 and 39 years, and 81.5% had a high educational level (university degree or above). Additionally, 32.7% of the participants were single. The association of demographic characteristics with awareness of colorectal cancer (CRC) is depicted in Table II. Among the participants, 61% of males and 54.9% of those aged 40 and above exhibited poor knowledge of CRC. No significant association was found between marital status and awareness of CRC. Participants with a lower educational level demonstrated poorer knowledge, whereas those with a higher educational level showed better awareness of CRC, with a statistically significant difference (p = 0.012). Table-II.

**Table-I:** The sociodemographic characteristics of participants in the survey on KAP of colon cancer among the people of Aljouf region.

Variables	Frequency n=422 (%)
*Gender*	
Male	252 (59.7)
Female	170 (40.3)
*Age*	
30 - 39 years	269 (63.7)
40 years old and above	153 (36.3)
*Marital status (Social status)*	
Single	138 (32.7)
Married	268 (63.5)
Other	16 (3.8)
*Education levels*	
Primary	1 (0.2)
Intermediate	8 (1.9)
Secondary (High school)	69 (16.4)
Graduated	244 (57.8)
Other	100 (23.7)
*Education levels*	
Lower education	78 (18.5)
Higher education	344 (81.5)

**Table-II:** Association of demographic characteristics with respect to awareness of colon cancer among the people of Aljouf region.

	Awareness of colon cancer		Chi-square χ^2^	
	High knowledge (n = 211) %	Poor knowledge (n = 211) %		P- value
*Gender*				
Male	98 (46.4)	154 (73.0)	30.892	< 0.00
Female	113 (53.6)	57 (27.0)		01
*Age*				
30 - 39 years	142 (67.3)	127 (60.2)	2.307	0.129
40 years old and above	69 (32.7)	84 (39.8)		
*Marital status (Social status)*				
Single	71 (33.6)	67 (31.8)		
Married	132 (62.6)	136 (64.5)	0.176	0.916
Other	8 (3.8)	8 (3.8)		
*Education levels*				
Primary	1 (0.5)	0 (0)		
Intermediate	1 (0.5)	7 (3.3)		
Secondary (High school)	27 (12.8)	42 (19.9)	18.171	0.001
Graduated	117 (55.5)	127 (60.2)		
Other	65 (30.8)	35 (16.6)		
*Education levels*				
Lower education	29 (13.7)	49 (23.2)	6.291	0.012
Higher education	182 (86.3)	162 (76.8)		

Table III highlights the association of demographic characteristics with knowledge of CRC signs and symptoms. Males (65.8%) showed poor knowledge regarding the signs and symptoms of CRC (p < 0.0001). Additionally, 54.2% of participants aged 30-39 years demonstrated high knowledge about CRC.

**Table-III:** The association of demographic characteristics with respect to knowledge of signs and symptoms of colon cancer among the people of Aljouf region.

	Knowledge of symptoms and signs of disease		Chi-square χ^2^	P- value
	High (n = 199) %	Poor (n = 223) %		
*Gender* Male Female	86 (43.2) 113 (56.8)	166 (74.4) 57 (25.6)	42.617	< 0.0001
*Age* 30 - 39 years 40 years old and above	146 (73.4) 53 (26.6)	123 (55.2) 100 (44.8)	15.088	< 0.0001
*Marital status (Social status)* Single Married Other	71 (35.7) 124 (62.3) 4 (2.0)	67 (30.0) 144 (64.6) 12 (5.4)	4.257	0.119
*Education levels*				
Primary	0 (0)	1 (0.4)		
Intermediate	2 (1.0)	6 (2.7)		
Secondary (High school)	28 (14.1)	41 (18.4)	20.308	< 0.0001
Graduated	103 (51.8)	141 (63.2)		
Other	66 (33.2)	34 (15.2)		
*Education levels*			2.903	
Lower education	30 (15.1)	48 (21.5)		0.088
Higher education	169 (84.9)	175 (78.5)		

Regarding early screening, 300 participants (71.1%) were aware that early checkups can reduce the risk of developing CRC, while 42 participants (10.0%) believed it to be irrelevant, and 80 participants (19.0%) were unsure. As for daily consumption of cereals and vegetables, 232 participants (55.0%) reported eating them daily, while 190 participants (45.0%) did not. Regarding the lack of exercise as a risk factor for CRC, 214 participants (50.7%) agreed, 85 participants (20.1%) disagreed, and 123 participants (29.1%) were uncertain. In terms of exercise habits, 200 participants (47.4%) exercised every day. The duration of daily exercise varied: 41 participants (9.7%) exercised for 0-15 minutes, 71 participants (16.8%) exercised for 15-30 minutes, 51 participants (12.1%) exercised for 30-60 minutes, and 38 participants (9.0%) exercised for an hour or more (Table IV).

**Table-IV:** Practices of people of Aljouf region regarding risk factors of colon cancer.

	Frequency n=422 (%)
*Does Early checkup reduce the chance of getting colon cancer?*	
Yes	300 (71.1)
No	42 (10)
Do not know	80 (19)
*Do you eat cereals and vegetables every day?*	
Yes	232 (55)
No	190 (45)
*Does Lack of exercise increases the risk of colon cancer?*	
Yes	214 (50.7)
No	85 (20.1)
Do not know	123 (29.1)
*Do you exercise every day?*	
Yes	200 (47.4)
No	222 (52.6)
*If you answered the previous question yes, how many minutes per day?*	
0 – 15 min.	41 (9.7)
15 – 30 min.	71 (16.8)
30 – 60 min.	51 (12.1)
An hour or more	38 (9)

## DISCUSSION

As far as the Aljouf region is concerned, this is the first study that has attempted to investigate the gaps in information regarding risk factors and early detection of colorectal cancer. Male participants had poorer knowledge of colorectal cancer (CRC) than female participants in our study. Carnahan and colleagues also found it to be similar.^11^ This inconsistency is worrying, especially when looking at the sex incidence of CRC, where the male-to-female ratio is higher. Appropriate and sex-specific public health education and CRC screening should be designed considering these results, especially effective ways for getting men more involved. Closing this knowledge gap could potentially help to diagnose CRC at an early and reduce mortality among men, who are generally at higher risk of developing CRC.

The respondents with the lowest education had significantly less knowledge than those with a higher education. This is consistent with other investigations showing that higher education individuals have more health awareness and access to health information.^12^,^13^ Health campaigns should be tailored to resonate with these lower-risk populations, who may have the most potential gains in earlier CRC detection and prevention with targeted health education. In Saudi Arabia social media is now widely available, where over 90% of the population have internet and handheld devices.^14^ This highlights an opening for health intervention, through which educational and screening messages could be broadcast on these platforms to potentially a more comprehensive range of audiences. Given the data presented here, social media may offer a way to increase awareness and utilization of screening programs, which could result in earlier diagnosis and better CRC outcomes.

The survey showed 50.7% knew the lack of exercise was a risk for developing CRC. In contrast, 84% of individuals recognised it in a comparable large cross-sectional study from Norway.^12^. Given that physical inactivity is a modifiable risk factor, increased understanding of its etiologic role in CRC could yield lifestyle interventions designed specifically to reduce the incidence of this cancer. In our survey, only 26% of participants identified diabetes mellitus as a CRC risk factor, aligning with a meta-analysis of 14 studies which highlighted that the risk of CRC for people with diabetes is about 38% greater than for non-diabetics, with a relative risk (RR) of 1.38 and a 95% CI of 1.26 to 1.51.^15^ Patients with diabetes develop CRC at a significantly higher rate; thus, publicizing this connection could prompt diabetic patients to undergo standard screening and take preventive actions.

In our study, 66% of the participants were aware that abdominal pain is a symptom of CRC. According to a survey conducted by Rashid et al., abdominal pain is listed as a common symptom of CRC.^16^ Recognizing symptoms like abdominal pain is essential for early diagnosis. To the extent that this leads to earlier medical consultation and diagnosis, with a subsequent improvement in public symptom awareness improving outcome.

In this study, 44.1% of participants were aware that weight loss is a warning sign of CRC. A study conducted in Lebanon showed 59.2% of participants were aware of that.^17^ which reflect a significant gap within our population. It indicates the need to increase the awareness of CRC signs and symptoms. In our study, only 40% agreed that the likelihood of having CRC increases if someone of relatives were diagnosed with colorectal cancer, it has been shown that environmental and genetic factors have associations with specific cancers.^18^ As such increasing the awareness and promoting early screening and prevention is needed, especially among those who have a family history.

### Study Strength & Limitations:

The strength of our study was that it has been based on a large survey with participants in all governorates in Aljouf region. Hence, is a representative study of a region which is seldom represented in surveys. We also used a detailed and comprehensive questionnaire to assess knowledge about risk factors, signs & symptoms and determining practices of people in Aljouf region regarding risk factors of CRC. Since our study was conducted in a specific geographic and cultural region, applicability of our findings to other populations may be affected. The responses can be influenced by recall bias.

## CONCLUSION

The population of the Aljouf region generally has low awareness of colorectal cancer (CRC), screening, and its risk factors. It is important to create health education programs aimed at raising awareness about the disease, its risk factors, and prevention. These programs would help promote awareness and reduce the incidence of colon cancer.

### Authors’ Contribution:

**HR:** Responsible and accountable for ensuring the accuracy and integrity of all steps from inception of research idea, IRB submission, questionnaire design, data collection, write up and final review of manuscript.

**FA, MSHA** and **HA:** Data analysis, data entry, literature review, interpretation of findings.

**MSA** and **MA:** Literature review, data collection. Critical |Analysis.
